# Nonlinear dynamics of curve-face gear transmission systems in two-parameter plane

**DOI:** 10.1371/journal.pone.0335920

**Published:** 2025-11-04

**Authors:** Jun Liu, Yanxia Zhang, Weimin Zhong, Fenxia Liu, Yinjing Yao

**Affiliations:** School of Automotive Engineering (SAIC-GM-Wuling Automotive Industry College), Guangxi Science & Technology Normal University, Laibin, Guangxi, China; Beijing Institute of Technology, CHINA

## Abstract

To systematically investigate the nonlinear dynamic characteristics of curve-face gear transmission systems and the influence of key parameters on their dynamic behavior, this study established a 6-degree-of-freedom bending-torsional coupling dynamic model. A two-parameter co-simulation numerical method was employed to explore the pattern types and existence regions of periodic motions within the two-parameter plane composed of key parameters. The transition mechanisms between non-impact vibrations and tooth-impact vibrations, as well as between adjacent fundamental periodic motions, were systematically revealed. Furthermore, the effects of parameter variations on the types and existence regions of periodic motion patterns were analyzed. The research results demonstrate that the transition between non-impact vibrations and tooth-impact vibrations occurs through grazing bifurcation, while transitions between adjacent tooth-impact motions are governed by period-doubling bifurcation. When the backlash exceeds 0.4125, variations in backlash values do not alter the types or existence regions of periodic motion patterns but only affect the displacement of the meshing pair. Increasing the meshing damping ratio significantly reduces the existence domains of tooth-impact periodic motions and chaotic motions in the two-parameter plane. This study provides theoretical foundations and practical references for the dynamic characteristic analysis and optimal design of curve-face gear transmission systems.

## 1 Introduction

With the increasing requirements for transmission accuracy, efficiency, and reliability in modern mechanical systems, traditional cylindrical gears and bevel gears have become insufficient to meet the variable speed ratio transmission demands under complex working conditions. Therefore, non-circular gears have gradually become a research hotspot. As a new type of non-circular gear transmission, the curve-face gear transmission system can achieve complex motion patterns and efficient transmission effects compared with traditional gears. The team from Chongqing University has conducted systematic theoretical and experimental research on the design method, meshing principle, motion characteristics, tooth surface contact analysis, and load capacity of the curve-face gear transmission system [1–4]. However, due to the complex geometric shape and time-varying meshing characteristics of the curve-face gear, its dynamic performance exhibits significant nonlinear characteristics. In recent years, the application of nonlinear dynamics theory in gear systems has gradually attracted significant attention from many scholars. In terms of dynamic modeling, Kahraman [5] established a torsional vibration dynamic model for compound gear trains to investigate their free vibration characteristics under different speed ratios. Raghothama [6] constructed an eight-degree-of-freedom dynamic analysis model for helical gears, which incorporated the stiffness of transmission shafts and bearings, and obtained the system’s dynamic response under the influence of transmission error. Lee [7] employed the finite element method to investigate the bending-torsional coupled vibration characteristics of gear-coupled rotor systems. During the variation of gear tooth meshing stiffness, certain vibration modes of the system exhibited bending-torsional coupled vibration characteristics.

The commonly used vibration analysis methods for gear transmission systems primarily include analytical methods, numerical methods, and experimental methods. Al-shyyab [8] employed the multi-scale harmonic balance method to solve the torsional vibration equations of multi-stage spur gear systems, analyzing the harmonic and sub-harmonic response characteristics of spur gears. Qiu [9] employed the multi-scale perturbation method to investigate the stability indices, stability regions, and stability performance of parallel-axis gear transmission systems. Zhou [10] derived the multi-degree-of-freedom vibration equations for gear-rotor systems and investigated the vibration response of spur gear-coupled rotor systems using the Runge-Kutta method. Kahraman [11] utilized experimentalmethods to study the influence patterns of parameters such as involute contact ratio on the vibration response of cylindrical gear transmission systems and planetary gear transmission systems. Liu [12] established a pure shear mechanical model of the low-range two-speed transmission system using the lumped parameter method and analyzed the variation of transmission errors between gears.

The structural parameters and configuration of gear systems exert a significant influence on their dynamic response. By establishing vibration equations for gear systems, computational analyses of vibration responses under different parameter conditions can be conducted to reveal the influence patterns of structural parameters, thereby enabling structural optimization design. Chaari [13] identified key factors affecting gear system vibration response, including gear tooth profile modification and eccentricity error, and obtained the influence patterns of various parameters on system dynamic characteristics. Inalpolat [14] established a dynamic model for multi-stage planetary gear transmission systems and analyzed the effects of planetary gear motion states on system vibration response.Chen [15] investigated the effects of friction and dynamic clearance on a multi-degree-of-freedom nonlinear dynamic gear transmission system characterized by time-varying stiffness. Cao [16] established a dynamic model of spur gears that considers the effects of force-dependent time-varying mesh stiffness, tooth backlash, and tooth profile deviation. Mo [17] investigated the effects of meshing damping, time-varying meshing stiffness, and load on the primary resonance of the system using numerical methods. Wang [18] analyzed the influence of temperature variation and time-varying stiffness coefficients on the bifurcation characteristics of gear systems, based on the principle of thermal deformation, taking into account the temperature effect and nonlinear parameters. Lin [19] established a nonlinear dynamic model for the torsional vibration of curve-face gear transmission systems, incorporating the influences of excitations from both the input and output shafts. Cai [20] proposed a generalized model based on the Lagrange Bond graphs. Vibration analysis of gears was conducted using this simulation model, and the numerical model was ultimately validated through experimental verification. Lin [21] developed a unified model for three configurations of compound motion curve-face gear pairs, investigated their dynamic characteristics, and verified the theoretical correctness through corresponding experimental studies. Lin [22] constructed a dynamic model and Lagrange Bond graph for eccentric curve-face gear transmission systems, accounting for the influence of cam effect inertia forces induced by eccentricity. The equations were derived based on the model, and the validity of the numerical analysis was confirmed through experimental investigations.



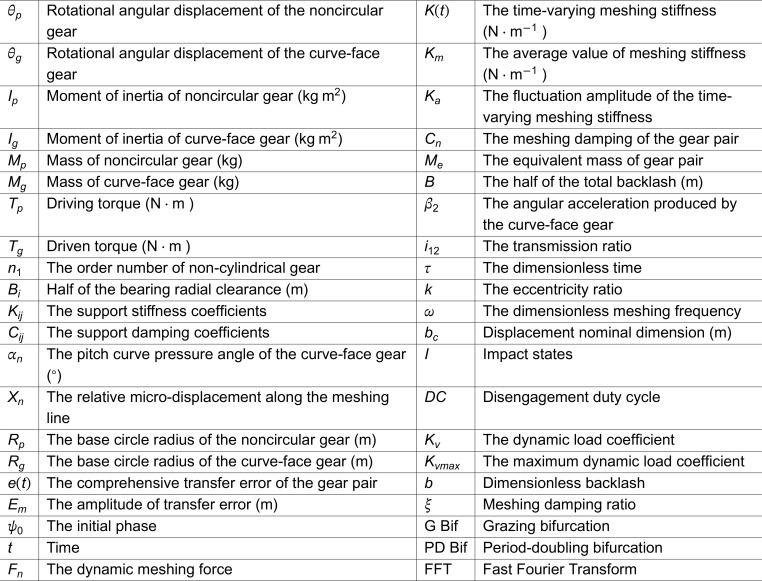



Existing studies have primarily focused on single-parameter simulation analysis. Although this method can reveal the effects of specific parameters on gear transmission systems, it is difficult to reflect the nonlinear dynamic characteristics of the system comprehensively. In contrast, two-parameter co-simulation analysis can more accurately reveal the formation mechanism of dynamic behaviors in gear transmission and provide a scientific basis for the matching design of global dynamic performance. In related studies, Farshidianfar [23] systematically investigated the bifurcation threshold characteristics of gear systems in parameter space using nonlinear parameters as control variables. Gou [24] conducted an in-depth analysis of the global dynamic characteristics of single-stage gear systems by establishing a gear meshing collision mapping model and revealed the dynamic evolution laws of the system based on basins of attraction and multi-parameter bifurcation diagrams. Yang [25] created a nonlinear dynamic model with twelve degrees of freedom for a multi-axis gear system and examined the system’s nonlinear characteristics within the parameter-state space. Shi [26] presents a method for computing coexistence dynamics within a two-parameter plane, taking into account multiple initial values through a gridding approach. Current research on the nonlinear dynamics of curve-face gears is limited and mainly restricted to single-parameter analysis [21–24], making it difficult to reveal the nonlinear dynamic characteristics from a system level.

The objective of this study is to employ a two-parameter co-simulation approach to investigate the types of periodic motions within the two-parameter plane and their corresponding existence regions for the curve-face gear system. Simultaneously, this study aims to elucidate the transition laws between non-impact vibrations and adjacent fundamental periodic impact vibrations, as well as between adjacent fundamental periodic motions. Furthermore, it analyzes how variations in parameters influence the types of periodic motions and their existence regions within the system. The structure of this study is divided into four parts. Sect 2 introduces the dynamic modeling and analysis methods of the curve-face gear system, detailing the modeling process as well as the impact state *I*, duty cycle *DC*, and maximum dynamic load $K_{vmax}$ during gear operation. Sect 3 addresses the system’s equations of motion by employing numerical methods and investigates the bifurcation and chaotic characteristics of the system under standard parameters. Additionally, based on the two-parameter co-simulation method, the types of periodic motions of the system and their existence regions within the parameter planes (ω,b) and (ω,ξ) are analyzed. Finally, Sect 4 summarizes the main conclusions of the study.

## 2 Dynamics model and methods

### 2.1 Dynamics model

**Fig 1:**
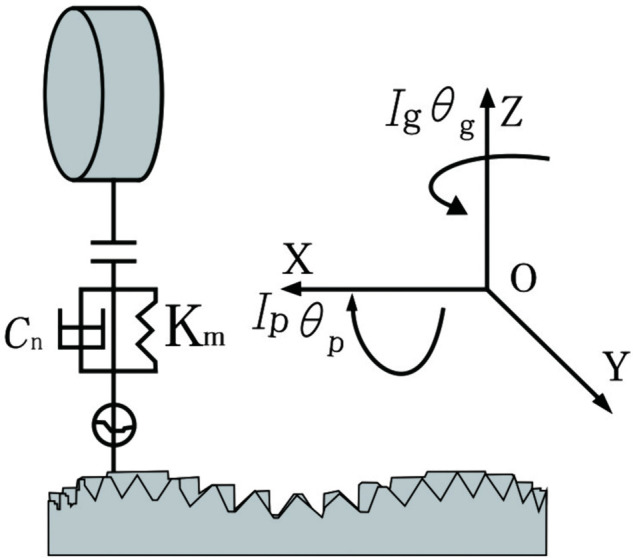
Dynamic model of curve-face gear transmission system.

A nonlinear dynamic model of the curve-face gear system is established as shown in Fig 1. The curve-face gear transmission system consists of a curve-face gear and a non-circular gear, where the non-circular gear serves as the driving gear and the curve-face gear acts as the driven gear. define *i*=*p*,*g*. *p* as the driving gear and *g* as the driven gear; *j*=*x*,*y*,*z*, denote the vibration displacement of gear *i* along each coordinate axis direction; $\theta_{i}$ denotes the rotational angular displacement of gear *i* around its respective axis; *M*_*i*_ and *I*_*i*_ respectively denote the concentrated mass and concentrated moment of inertia of gear *i*; *T*_*i*_ denotes the torque applied to gear *i*; The rolling bearings supporting the gears are equivalent to linear springs and linear dampers in the *Y* and *Z* coordinate directions. The support stiffness coefficients are denoted as *K*_*ij*_, and the support damping coefficients are denoted as *C*_*ij*_; Ω denotes the meshing frequency of the gear pair.

The curve-face gear transmission system has 6 degrees of freedom, and its generalized coordinates can be expressed as $(\text{Yp},\text{Zp},\text{Yg},\text{Zg},\theta_{p},\theta_{g}){\;}^{T}$.

The radial clearance of the rolling bearing is analyzed in the *Y* and *Z* coordinate directions, which can be represented using the nonlinear clearance function from equation:

$$f(Y_{i},B_{i})=\left\{ \begin{array}{c} Y_{i}-B_{i},(Y_{i}>B_{i})\\ 0,(-B_{i}\leq Y_{i}\leq B_{i})\\ Y_{i}+B_{i},(Y_{i}>B_{i}) \end{array}\right.,f(Z_{i},B_{i})=\left\{ \begin{array}{c} Z_{i}-B_{i},(Z_{i}>B_{i})\\ 0,(-B_{i}\leq Z_{i}\leq B_{i})\\ Z_{i}+B_{i},(Z_{i}>B_{i}) \end{array}\right.$$ (1)

where *B*_*i*_ represents half of the bearing radial clearance.

The relative micro-displacement *X*_*n*_ along the meshing line due to transmission error and vibration is expressed as:

$$X_{n}=(Y_{p}-Y_{g})\cos\alpha_{n}+(Z_{p}-Z_{g})\sin\alpha_{n}+(R_{p}\theta_{p}-R_{g}\theta_{g})\cos\alpha_{n}-e(t)$$ (2)

where $\alpha_{n}$ is the pitch curve pressure angle of the curve-face gear. *R*_*p*_ is the base circle radius of the non-circular gear, *R*_*g*_ is the base circle radius of the curve-face gear. *e*(*t*) is the comprehensive transfer error of the gear pair, and can be expressed by the equation $e(t)=E_{m}\cos(\Omega t\;+\;\psi_{0})$. In the equation, *E*_*m*_ represents the amplitude of transfer error, $\psi_{0}$ represents the initial phase, and *t* represents the time.

The dynamic meshing force *F*_*n*_ of the curve-face gear transmission system and its component forces *F*_*i*_ along each coordinate axis direction are expressed as:

$$\left\{ \begin{array}{c} F_{n}=C_{n}{\dot{X}}_{n}+K(t)f(X_{n},B)\\ F_{y}=F_{n}\cos\alpha_{n}\\ F_{z}=F_{n}\sin\alpha_{n} \end{array}\right.$$ (3)

Where *K*(*t*) is the time-varying meshing stiffness of the gear pair. It can be expressed as the equation $K(t)=K_{m}(1\;+\;K_{a}\cos(\Omega t\;+\;\psi_{0}))$, where *K*_*m*_ is the average value of meshing stiffness, *K*_*a*_ is the fluctuation amplitude of the time-varying meshing stiffness, *C*_*n*_ is the meshing damping of the gear pair, and its expression is $C_{n}=2\xi \sqrt{K_{m}M_{e}}$, where ξ is the relative damping ratio of the meshing gear pair, and *M*_*e*_ is the equivalent mass of the gear pair.

In Eq (3), the function of gear backlash is represented by *f*(*X*_*n*_,*B*) as the following equation:

$$f(X_{n},B)=\left\{ \begin{array}{c} X_{n}-B,(X_{n}>B)\\ 0,(-B\leq X_{n}\leq B)\\ X_{n}+B,(X_{n}>B) \end{array}\right.$$ (4)

where *B* is the half of the total backlash.

The time-varying meshing stiffness is a critical parameter in the dynamic equations of gear systems. Its magnitude is related to the contact ratio, and when the contact ratio is non-integer, the mesh stiffness becomes a periodic function of the rotation angle. In this paper, the ISO draft formula for tooth stiffness calculation is adopted to approximate the tooth stiffness of the curve-face gear [19]. The approximate value of the tooth stiffness per unit width is:

$$\overset{´}{C}=\frac{1}{q}\cos\alpha_{n}\cos\beta_{g}$$ (5)

$$\begin{array}{c} q=0.04723+\frac{0.15551}{z_{v1}}+\frac{0.25791}{z_{v2}}-0.006325x_{1}-0\mathrm{.11654}\frac{x_{1}}{z_{v1}}\\ -0.00193x_{2}-0.24188\frac{x_{2}}{z_{v2}}+0\mathrm{.00529}x_{1}^{2}+0.00529x_{2}^{2} \end{array}$$ (6)

Where $\beta_{g}$ is the helix angle of helical gears, taken as 0, $z_{v1}$ and $z_{v2}$ are the number of teeth in the noncircular gear and curve-face gear, and *x*_1_ and *x*_1_ are the modification coefficients of the noncircular gear and curve-face gear, taken as 0. After considering the tooth width factor, the tooth stiffness of the orthogonal curve-face gear is:

$$k_{2}=\frac{1}{q}B\cos\alpha_{n}$$ (7)

The meshing stiffness can be approximated as the stiffness in the double-tooth meshing zone. The meshing stiffness is expressed as:

$$K(t)=1.5\overset{´}{k}=1.5\frac{k_{1}k_{2}}{k_{1}+k_{2}}$$ (8)

where *k*_1_ is the tooth stiffness of the noncircular gear, *k*_2_ is the tooth stiffness of the curve-face gear, and $k_{1}=k_{2}$, $\overset{´}{k}$ is the meshing stiffness of the single-tooth meshing zone. Taking into account the problem of unequal load distribution, a common practice is to utilize 1.5 times the stiffness of single-tooth meshing as the meshing stiffness for the zone with double-tooth meshing.

Based on Newton’s second law, the 6-degree-of-freedom motion differential equations for the curve-face gear transmission system are established as follows [20]:

$$\left\{ \begin{array}{c} M_{p}{\ddot{Y}}_{p}+C_{py}{\dot{Y}}_{p}+K_{py}f(Y_{p},B_{p})=-F_{y}-\lambda \mu F_{n}\sin\alpha_{n}\\ M_{p}{\ddot{Z}}_{p}+C_{pz}{\dot{Z}}_{p}+K_{pz}f(Z_{p},B_{p})=F_{z}-\lambda \mu F_{n}\cos\alpha_{n}\\ I_{p}{\ddot{\theta }}_{p}=T_{p}-F_{y}R_{p}-\lambda \mu F_{n}L_{p}\sin\alpha_{n}\\ M_{g}{\ddot{Y}}_{g}+C_{gy}{\dot{Y}}_{g}+K_{gy}f(Y_{g},B_{g})=F_{y}+\lambda \mu F_{n}\sin\alpha_{n}\\ M_{g}{\ddot{Z}}_{g}+C_{gz}{\dot{Z}}_{g}+K_{gz}f(Z_{g},B_{g})=-F_{z}+\lambda \mu F_{n}\cos\alpha_{n}\\ I_{g}{\ddot{\theta }}_{g}=-(T_{g}-I_{g}\beta_{2})+F_{y}R_{g}+\lambda \mu F_{n}R_{g}\sin\alpha_{n} \end{array}\right.$$ (9)

where μ is the coefficient of sliding friction; *λ* is the friction force direction function, and its expression is $\lambda =\text{sgn}(L_{P}\;-\;R_{b1}\tan\alpha_{n})$, *L*_*p*_ is the time-varying friction arm direction and its expression is $L_{p}=\sqrt{R_{ap}^{2}\;+\;R_{bp}^{2}}\;-\;\varepsilon P_{bp}\;+\;nR_{bp}t$, where *R*_*ap*_ is the addendum circle radius of the non-circular gear, *ε* is the contact ratio of the curve-face gear pair, *P*_*bp*_ is the base circle pitch, and *n* is the rotational speed. $\beta_{2}$ is the angular acceleration produced by the curve-face gear, whose expression is $T_{p}=(T_{g}\;-\;I_{g}\beta_{2})/i_{12}$, where *i*_12_ is the transmission ratio of the curve-face gear transmission system.

Since the curve-face gear transmission system is a variable transmission ratio system, the expression for the transmission ratio *i*_12_ is:

$$i_{12}=\frac{R(1-k\cos n_{1}\theta_{p})}{a(1-k^{2})}$$ (10)

where *R* is the radius of the curve-face gear pitch curve, a represents the semi-major axis, *k* is the eccentricity ratio, and *n*_1_ and $\theta_{p}$ are the order and the rotation angle of the noncircular gear.

The nominal dimension is defined as *b*_*c*_, the inherent frequency as $\omega_{n}=\sqrt{K_{m}/M_{pg}}$, dimensionless parameters, $y_{i}=Y_{i}/b_{c}$, $z_{i}=Z_{i}/b_{c}$, $x_{n}=X_{n}/b_{c}$, $\vartheta_{i}=\theta_{i}/b_{c}$, *b* = *B*/*b*_*c*_, $b_{i}=B_{i}/b_{c}$, $e_{m}=E_{m}/b_{c}$, $\tau =\omega_{n}t$, $\omega =\Omega /\omega_{n}$, $e(\tau )=e(t)/b_{c}$, (i=p,g), where *τ* is the dimensionless time, and ω is the dimensionless meshing frequency.

The above equations of dimensionless parameters are plugged into Eq (9), and the following differential Eq (11) of dimensionless motion of the system is obtained.

$$\left\{ \begin{array}{c} {\ddot{y}}_{p}+2\xi_{py}{\dot{y}}_{p}+2(\cos\alpha_{n}+\lambda \mu \sin\alpha_{n})\xi_{hp}{\dot{x}}_{n}+k_{py}f(y_{p},b_{p})+(\cos\alpha_{n}+\lambda \mu \sin\alpha_{n})k_{hp}f(x_{n},b)=0\\ {\ddot{z}}_{p}+2\xi_{pz}{\dot{z}}_{p}-2(\sin\alpha_{n}-\lambda \mu \cos\alpha_{n})\xi_{hp}{\dot{x}}_{n}+k_{pz}f(z_{p},b_{p})-(\sin\alpha_{n}-\lambda \mu \cos\alpha_{n})k_{hp}f(x_{n},b)=0\\ {\ddot{\vartheta }}_{p}+2(R_{p}\cos\alpha_{n}-\lambda \mu l_{1}\sin\alpha_{n})\xi_{p}{\dot{x}}_{n}+(R_{p}\cos\alpha_{n}-\lambda \mu l_{1}\sin\alpha_{n})k_{p}f(x_{n},b)=f_{p}-f_{e}\\ {\ddot{y}}_{g}+2\xi_{gy}{\dot{y}}_{g}-2(\cos\alpha_{n}+\lambda \mu \sin\alpha_{n})\xi_{hg}{\dot{x}}_{n}+k_{gy}f(y_{g},b_{g})-(\cos\alpha_{n}+\lambda \mu \sin\alpha_{n})k_{hg}f(x_{n},b)=0\\ {\ddot{z}}_{g}+2\xi_{gz}{\dot{z}}_{g}+2(\sin\alpha_{n}-\lambda \mu \cos\alpha_{n})\xi_{hg}{\dot{x}}_{n}+k_{gz}f(z_{g},b_{g})+(\sin\alpha_{n}-\lambda \mu \cos\alpha_{n})k_{hg}f(x_{n},b)=0\\ {\ddot{\vartheta }}_{g}-2R_{g}(\cos\alpha_{n}+\lambda \mu \sin\alpha_{n})\xi_{g}{\dot{x}}_{n}-R_{g}(\cos\alpha_{n}+\lambda \mu \sin\alpha_{n})k_{g}f(x_{n},b)=f_{g}-f_{e} \end{array}\right.$$ (11)

where $k_{ij}=\Omega_{ij}^{2}/\omega_{n}^{2}$, $\xi_{ij}=C_{ij}/(2M_{i}\omega_{n})$, $\xi_{hi}=C_{n}/(2M_{i}\omega_{n})$, $k_{hi}=K(\tau )/(M_{j}\omega_{n}^{2})$, $f_{a}=\beta_{2}R_{g}/(b_{c}\omega_{n}^{2})$, $f_{e}=e_{m}\omega^{2}\cos(\omega \tau \;+\;\psi_{0})$, $f_{g}=T_{g}R_{g}/(I_{g}b_{c}\omega_{n}^{2})$, $f_{p}=T_{p}R_{p}/(I_{p}b_{c}\omega_{n}^{2})$, ξ tooth meshing damping ratio in gear pair.

The functions of non-dimensional backlash and non-dimensional bearing radial clearance are represented by Eq (12) and Eq (13), respectively.

$$f(x_{n},B)=\left\{ \begin{array}{c} x_{n}-b,(x_{n}>b)\\ 0,(-b\leq x_{n}\leq b)\\ x_{n}+b,(x_{n}>b) \end{array}\right.$$ (12)

$$f(y_{i},b_{i})=\left\{ \begin{array}{c} y_{i}-b_{i},(y_{i}>b_{i})\\ 0,(-b_{i}\leq y_{i}\leq b_{i})\\ y_{i}+b_{i},(y_{i}>b_{i}) \end{array}\right.,f(z_{i},b_{i})=\left\{ \begin{array}{c} z_{i}-b_{i},(z_{i}>b_{i})\\ 0,(-b_{i}\leq z_{i}\leq b_{i})\\ z_{i}+b_{i},(z_{i}>b_{i}) \end{array}\right.,(i=p,g)$$ (13)

The primary design parameters of the curve-face gear transmission system under study are as follows: modulus *m* = 4mm, number of teeth for non-cylindrical gears *z*_*p*_ = 18, number of teeth for curve-face gear *z*_*g*_ = 36, order of non-cylindrical gears *n*_1_ = 2, order of curve-face gear *n*_2_ = 4, and normal pressure angle $\alpha_{n}$ = 20^°^. The selection of standard dynamical parameters is provided in Table 1.

**Table 1:** Standard parameters of the curve-face gear transmission system.

Parameter	Value	Parameter	Value
Gear backlash2*B*/m	$100\times 10^{-6}$	Driven torque *T*_*p*_ (N·m )	150
Bearing radial clearance 2*B*_*i*_/m	$100\times 10^{-6}$	Average meshing stiffness *K*_*m*_($N\cdot m^{-1}$)	$4.2\times 10^{8}$
Comprehensive transmission error *E*_*m*_/m	$20\times 10^{-6}$	Meshing damping ratio ξ	0.7
Displacement nominal dimension *b*_*c*_/m	$50\times 10^{-6}$	Time-varying meshing stiffness amplitude *K*_*a*_	0.2

### 2.2 Methods

#### 2.2.1 Impact state.

**Fig 2:**
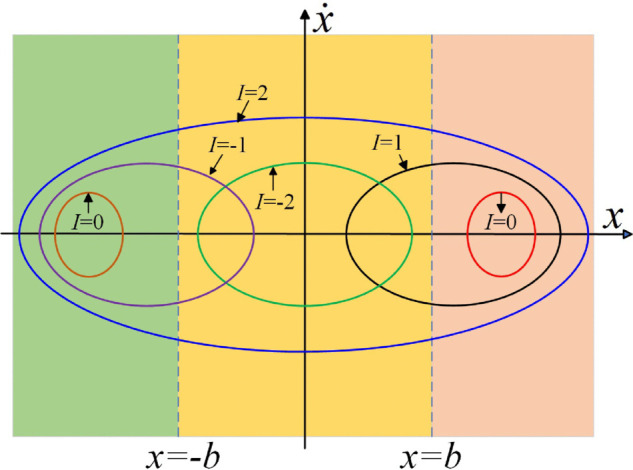
Distribution of the system’s phase trajectories on the plane.

As illustrated in Fig 2, the backlash delineates the phase plane into three distinct regions. During the meshing process of the system, the motion trajectory may manifest five impact states, referred to as *I*. Specifically:

1. *I* = 0: Non-impact state.
2. *I* = 1: Tooth surface impact state.
3. *I* = −1: Tooth back impact state.
4. *I* = 2: Double-sided impact state.
5. *I* = −2: Complete disengagement state.

By combining the above 5 types of impact states, the motion pattern *I*/*n*T of the gear pair is obtained, where *I* represents the meshing impact state during gear meshing, *n* denotes the number of periods of the system’s meshing motion, and T = 2*π*/ω represents the meshing period time of the system.

To characterize the disengagement severity of the gear pair during meshing impact processes, the disengagement duty cycle (*DC*) is utilized. *DC* is defined as the ratio of the duration of disengagement phenomena to the total operational time during gear rotation. In the non-impact state, *DC* = 0.0. In single-sided impact (tooth surface impact, tooth back impact) and double-sided impact states, 0 < *DC* < 1. A higher *DC* value indicates a longer duration of disengagement and a more severe disengagement condition in the gear pair.

Due to manufacturing and assembly errors, elastic deformation of gear teeth, and the time-varying characteristics of meshing stiffness, coupled with the alternating occurrence of single-tooth and double-tooth meshing in gear pairs, additional dynamic loads or impacts are generated during the meshing process [27,28]. The dynamic load coefficient ($K_{v}$) is commonly used to evaluate the deviation between the actual dynamic load and the theoretical load in gear dynamics research. Since the dynamic load during the meshing process varies over time, continuous sampling of the dynamic load coefficient for each meshing cycle (T = 2*nπ*/ω) is conducted. The maximum value in the obtained dataset is the maximum dynamic load coefficient ($K_{vmax}$) under operating conditions. The larger the ($K_{vmax}$) value, the greater the alternating impact stress and the more severe the impact noise.

#### 2.2.2 Co-simulation method.

**Fig 3:**
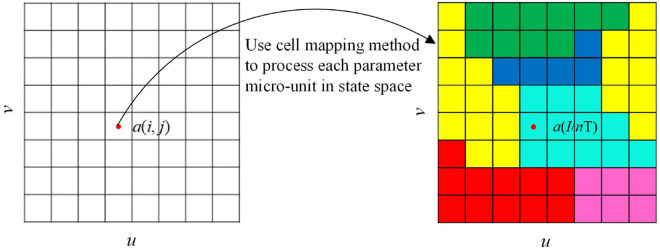
Schematic diagram of two-parameter co-simulation.

In the schematic diagram of two-parameter co-simulation presented in Fig 3, the two-parameter co-simulation is developed based on cell mapping. This method involves selecting two critical parameters from the system parameters to construct a two-parameter plane. The system parameters, denoted as *u* and *v*, form *a* (*u*, *v*) parameter plane that is divided into a finite number of grids. By calculating the periodicity and impact counts of stable phase trajectories at the center points *a*(*i*, *j*) of each grid, the motion state of the system within that grid can be determined. The motion state of phase trajectories at each point on the parameter plane is computed, and grids exhibiting the same motion state are marked with identical colors and symbols. This process elucidates the pattern types and distribution rules of periodic motion across the entire two-parameter plane. In this study, a 400 × 400 grid system is employed for calculations.

## 3 Dynamic analyses

### 3.1 Nonlinear dynamics under standard parameters

**Fig 4:**
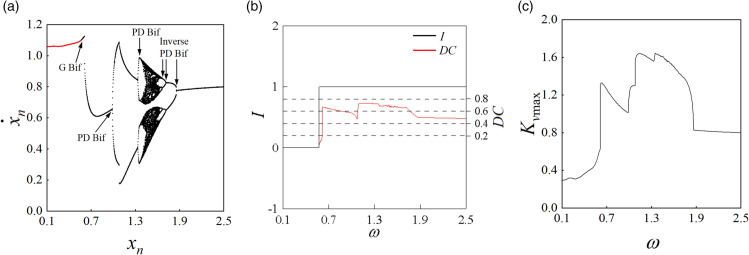
(a) Bifurcation diagram of mesh displacement; (b) I: DC diagram; (c) $K_{vmax}$ diagram.

The fourth-order variable-step Runge-Kutta method is utilized to solve the motion differential equations of the curve-face gear transmission system. Various research tools, including bifurcation diagrams, impact state diagrams, disengagement duty cycle diagrams, phase diagrams, time history diagrams, dynamic load coefficient diagram, and Poincaré maps, are employed to analyze the nonlinear dynamic characteristics of the system under varying meshing excitation frequencies, thereby revealing the transition laws of periodic motion. With the standard parameters outlined in Table 1, the dimensionless meshing frequency ω is selected as the control parameter, varying within the range [0.1, 2.5]. Through numerical simulations, the meshing displacement bifurcation diagram, impact states: disengagement duty cycle (*I*: *DC*) diagram, and maximum dynamic load coefficient ($K_{vmax}$) diagram of the system as illustrated in Fig 4.

**Fig 5:**
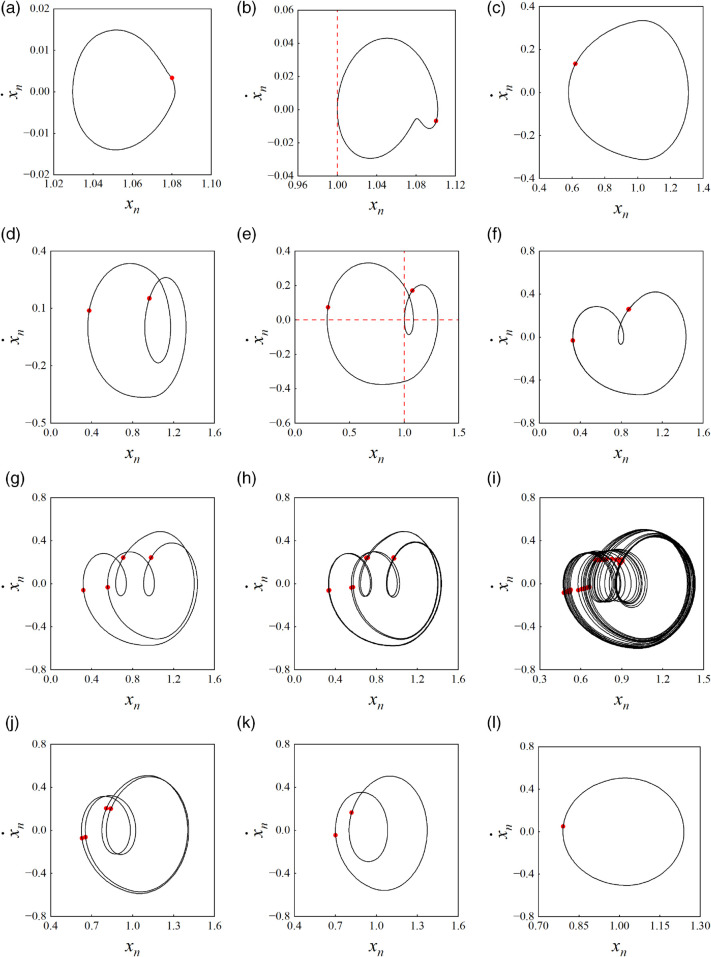
Superpositions of the phase diagram and Poincaré mapping of the gear pair: (a) ω = 0.48 1-0-0, (b) ω = 0.569 grazing, (c) ω = 0.86 1-1-0, (d) ω = 1.02 2-2-0, (e) ω = 1.072 grazing, (f) ω = 1.25 2-1-0, (g) ω = 1.37 4-3-0, (h) ω = 1.384 8-6-0, (i) ω = 1.53 chaotic, (j) ω = 1.7 4-3-0, (k) ω = 1.81 2-2-0, (l) ω = 2.24 1-1-0.

**Fig 6:**
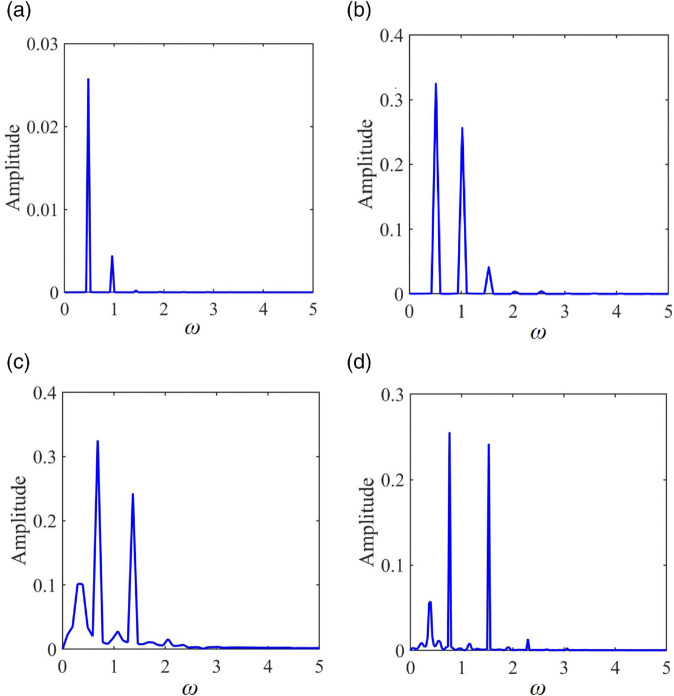
FFT spectrum: (a) ω = 0.48 1-0-0, (b) ω = 1.02 2-2-0, (c) ω = 1.37 4-3-0, (d) ω = 1.53 chaotic.

Fig 4(a) presents the bifurcation diagram of the curve-face gear transmission system. In this diagram, red indicates the non-impact state (*I* = 0), while black represents the tooth surface impact state (*I* = 1). It is evident that when ω < 0.569, the system exhibits 1-0-0 periodic motion with impact state *I* = 0. In this regime, the gear pair operates in a state without impact, characterized by a disengagement duty cycle (*DC*) of 0, which implies that no tooth surface separation occurs during the meshing period, allowing for smooth system operation. The $K_{vmax}$ displays a gradual upward trend. As illustrated in Fig 5(a) for ω = 0.48, the superposition of the phase diagram and Poincaré map for the 1-0-0 periodic motion is shown, where • denotes points formed by the trajectory crossing the Poincaré section. A single periodic mapping point is generated when the trajectory intersects the Poincaré section. As shown in Fig 6(a), the FFT spectrum appears only at the meshing frequency ω. As the system crosses ω = 0.569, a grazing bifurcation (which is marked as “G Bif” in Fig 4) occurs, leading to a transition from 1-0-0 periodic motion to 1-1-0 periodic motion via this bifurcation, with the impact state changing from *I* = 0 to *I* = 1 (from non-impact to tooth surface impact). This transition corresponds to the phenomenon of "tooth surface separation-contact-tooth surface separation" within the meshing period. Simultaneously, $K_{vmax}$ experiences a sudden change, accompanied by intensified impact vibration noise and instability in system operation. Fig 5(b) illustrates the phase diagram at the onset of grazing bifurcation, where the Poincaré section exhibits one stable periodic mapping point, indicating that the gear pair experiences one tooth surface impact within a meshing period. At the grazing bifurcation point $\;x_{n}=0.0$ and ${\dot{x}}_{n}=0.0$. Grazing causes the system to jump around the bifurcation critical value of the control parameter ω = 0.66, resulting in a sharp increase in both the disengagement duty cycle (*DC* = 0.66) and the maximum dynamic load coefficient ($K_{vmax}$ = 1.311). The *I* remains unchanged.

After surpassing the jump threshold, the system stabilizes into a 1-1-0 periodic motion, with both *DC* and $K_{vmax}$ rapidly decreasing. Fig 5(c) illustrates the phase diagram of the 1-1-0 periodic motion. As ω increases further, the system crosses the period-doubling bifurcation (which is marked as “PD Bif” in Fig 4) threshold at ω = 0.988, transitioning from 1-1-0 to 2-2-0 periodic motion (as shown in Fig 5(d) for ω = 1.02). In this state, two tooth surface impacts occur, with the trajectory crossing the Poincaré section twice, resulting in two stable discrete points. As shown in Fig 6(b), the FFT spectrum exhibits subharmonic ω/2 and its harmonics *n*ω/2 (where *n* is an integer).

At ω = 1.072, a grazing bifurcation transitions the system from 2-2-0 to 2-1-0 motion. The *DC* and $K_{vmax}$ increase linearly, which reduces the load capacity of the gear pair and amplifies vibration noise. Fig 5(e) illustrates the phase diagram corresponding to this grazing bifurcation. The transition process from 2-2-0 to 2-1-0 is depicted in Fig 5(d), 5(e), and 5(f). At ω = 1.33, period-doubling bifurcation drives the system from 2-1-0 to 4-2-0 motion. During this bifurcation, the period number (*n*) and tooth surface impact count (*p*) double, while $K_{vmax}$ continues to rise, exacerbating vibration noise. At ω = 1.354, the system undergoes a grazing bifurcation, transitioning from 4-2-0 motion to 4-3-0 motion. Fig 5(g) presents the phase diagram for 4-3-0 motion at ω = 1.37, featuring four periodic mapping points on the Poincaré section. As shown in Fig 6(c), the spectral lines of the FFT spectrum are distributed at discrete points of *n*ω/4. At ω = 1.37, the system enters 8-6-0 periodic motions, respectively, through a continuous series of period-doubling bifurcations. Fig 5(h) displays the superposition of the phase diagram and Poincaré map for 8-6-0 periodic motion, revealing eight stable periodic mapping points on the Poincaré section. As ω continues to increase, the system enters chaotic motion at ω = 1.396. Throughout this transition, the impact state of the system remains unchanged. Fig 5(i) shows the superposition of the phase diagram and Poincaré map for chaotic motion, where the phase diagram features numerous intersecting closed or open curves, and the Poincaré section exhibits many scattered discrete points. As shown in Fig 6(d), continuous frequency components appear in the FFT spectrum. As ω continues to rise, the system experiences continuous inverse period-doubling bifurcations, gradually transitioning from chaotic motion into 8-6-0, 4-3-0, and 2-2-0 subharmonic motions at ω = 1.642, ω = 1.672, and ω = 1.714, respectively. At ω = 1.864, the system stabilizes into a 1-1-0 periodic motion. The *DC*, $K_{vmax}$, and the number of motion periods all exhibit a rapid decreasing trend. Fig 4(j), 4(k), and 4(l) illustrate the superposition of phase diagrams and Poincaré maps for the 4-3-0, 2-2-0, and 1-1-0 motions, respectively.

### 3.2 Pattern types and transition laws of periodic motion in (*ω*omega, *b*) parameter plane

**Fig 7:**
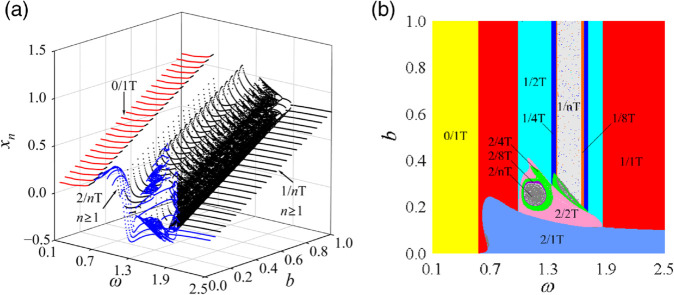
Meshing motion law diagram of the system in the (ω, b) parameter plane: (a) relative micro-displacement bifurcation diagram of gear pair, (b) pattern types and distribution regions of periodic motions.

This section employs a two-parameter co-simulation to investigate the nonlinear dynamic characteristics of curve-face gear transmission systems. By taking dimensionless meshing frequency (*ω*) and dimensionless backlash (*b*) as key parameters, where *ω* ∈ [0.1, 2.5] and *b*∈ [0.0, 1.0] while keeping other parameters constant, the pattern types, distribution regions, and transition laws of periodic motions in the (*ω*, *b*) parameter plane were identified through two-parameter co-simulation, as illustrated in Fig 7. Fig 7(a) presents a three-dimensional bifurcation diagram of the system’s meshing displacement as parameters vary. The blue color indicates that the system’s impact state is *I* = 2, characterized by double-sided impact. It is observed that the non-impact state occurs only in the low-frequency region, while the tooth surface impact state exists in the high-frequency region with larger *b* values. Conversely, double-sided impact primarily occurs in the high-frequency region with smaller backlash values. The system exhibits various bifurcation behaviors during meshing motion, including grazing bifurcation, period-doubling bifurcation, and inverse period-doubling bifurcation. Furthermore, the displacement of the meshing pair increases with the increasing *b* value.

Fig 7(b) illustrates the distribution of periodic motions within the (*ω*, *b*) parameter plane, providing an intuitive representation of the types, distribution areas, and variation laws of these motions. In this parameter plane, *I*/*n*T is utilized to identify the impact state at the backlash, with distinct colors representing various types of motion patterns. The primary periodic motions present in the (*ω*, *b*) parameter plane include: 0/1T periodic motion, 1/1T periodic motion, tooth surface impacts indicated by different colors, double-sided impact subharmonic periodic motion, double-sided impact quasi-chaotic motion denoted by 2/*n*T in dark gray regions, and tooth surface impact quasi-chaotic motion represented by 1/*n*T in light gray regions. When *b* < 0.4125, a significant number of double-sided impact periodic motions and chaotic behaviors emerge in the (*ω*, *b*) parameter plane, leading to extreme instability in system operation characterized by pronounced vibrations and noise, alongside complex dynamic behaviors. As *b* increases, particularly within the range of [0.4125, 1.0], the system ceases to exhibit double-sided impact periodic motions in the (*ω*, *b*) parameter plane; rather, only tooth surface impact periodic motions occur, resulting in a more stable operation with reduced vibrations and noise, alongside improved performance metrics. At lower frequencies, the system demonstrates 0/1T non-impact periodic motion. As *ω* increases, a grazing bifurcation is observed, with the periodic motion shifting from non-impact 0/1T motion to tooth surface impact 1/1T motion. Subsequently, a period-doubling bifurcation occurs, resulting in a transition of the periodic motion from 1/1T state to 2/2T subharmonic periodic motion. Subsequently, a series of continuous period-doubling bifurcations takes place, leading to higher-periodic motions such as 1/4T, 1/8T, and so forth, ultimately culminating in chaotic motion. As *ω* further increases, this chaotic motion degenerates into lower-periodic motions, including 1/8T, 1/4T, 1/2T, and returning to 1/1T through a series of continuous inverse period-doubling bifurcations. Moreover, when the parameter *b* is within the range [0.4125, 1.0], variations in *b* do not affect the periodic motion characteristics of the system; rather, variations in *b* only influence the displacement of the meshing pair. The grazing bifurcation value, period-doubling bifurcation value, and inverse period-doubling bifurcation value remain unchanged with respect to *b*, indicating that the system behaves similarly to a linear system.

**Fig 8:**
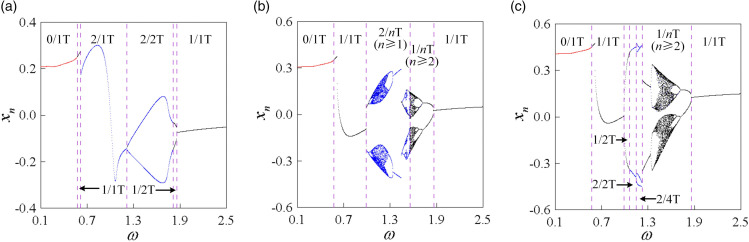
Single-parameter bifurcation diagrams of mesh displacement under different dimensionless backlash b: (a) b = 0.15, (b) b = 0.25, (c) b = 0.35.

To investigate the global bifurcation characteristics of the system more clearly, single-parameter bifurcation diagrams were obtained by traversing the two-parameter plane transversely at *b* = 0.15, 0.25, and 0.35, as illustrated in Fig 8.

Fig 8(a) presents the single-parameter bifurcation diagram of the system at *b* = 0.15. In the low-frequency region, the system exhibits non-impact 0/1T motion. As *ω* increases, the system transitions from a non-impact state to a double-sided impact state, eventually entering a stable tooth surface impact state, represented as 0/1T →1/1T → 2/1T → 2/2T → 1/2T → 1/1T. Fig 8(b) presents the global single-parameter bifurcation diagram of the system at *b* = 0.25. Compared with the bifurcation diagram at *b* = 0.15, the non-impact state interval in the low-frequency region remains unchanged, but the double-sided impact state interval contracts and gradually transforms into a tooth surface impact state, with the tooth surface impact state interval expanding. However, high-period double-sided impact motions such as period 4 and 8 appear in the double-sided impact state interval. Fig 8(c) depicts the global single-parameter bifurcation diagram at *b* = 0.35. As *b* increases, the non-impact state region at low frequencies remains unchanged, but the double-sided impact state interval significantly contracts and gradually transforms into a tooth surface impact state, resulting in a noticeable expansion of the tooth surface impact state interval. However, the motions in the double-sided impact state interval degenerate into low-period double-sided impact motions such as 2/4T and 2/2T.

**Fig 9:**
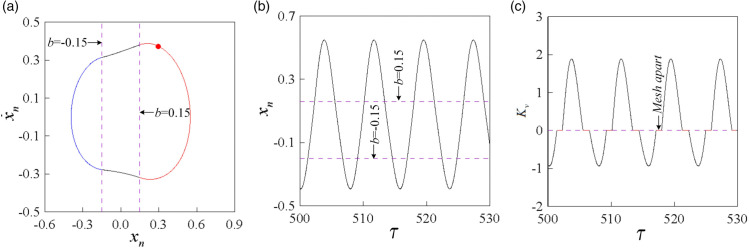
1-1-1 double-sided impact motion, when b = 0.15, ω = 0.82: (a) superposition of phase diagram and Poincaré map, (b) time history diagram, (c) dynamic load coefficient diagram $K_{v}$.

**Fig 10:**
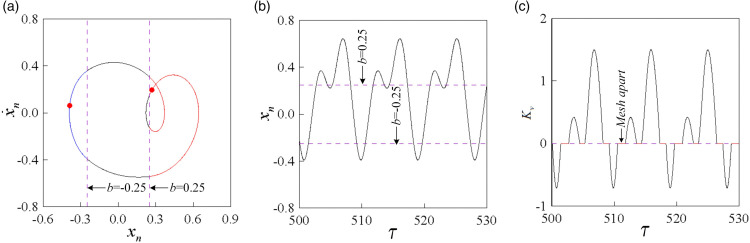
2-2-1 double-sided impact motion, when b = 0.15, ω = 0.82: (a) superposition of phase diagram and Poincaré map, (b) time history diagram, (c) dynamic load coefficient diagram $K_{v}$.

**Fig 11:**
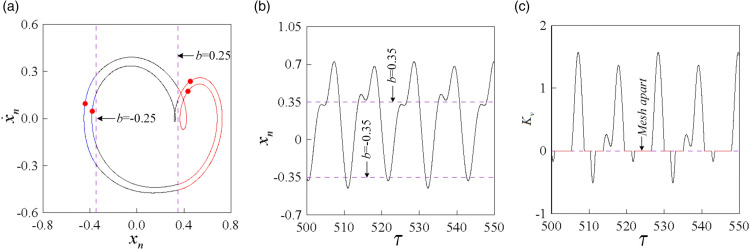
4-4-2 double-sided impact motion, when b = 0.15, ω = 0.82: (a) superposition of phase diagram and Poincaré map, (b) time history diagram, (c) dynamic load coefficient diagram $K_{v}$.

Figs 9, 10, and 11 depict the phase diagram and Poincaré map superposition, along with the time history and dynamic load coefficient diagram for the double-sided impact motion under different dimensionless backlash *b* and dimensionless meshing frequency. By integrating the phase diagram with the time history diagram, one can observe the number of motion cycles (*n*), the number of tooth face impacts (*p*), and the number of tooth back impacts (*q*) of the gear pair, thereby determining the impact state (*I*) of the gear. The irregularity of the dynamic load coefficient ($K_{v}$) is notable, as it can exhibit positive, zero, and negative values. When $K_{v}$ reaches zero, the gear teeth engaged in meshing experience alternating dynamic loads. Prolonged operation of the system within this parameter range can result in fatigue fractures at the tooth root or damage to the tooth surface. Therefore, it is evident that gear backlash significantly influences both the characteristics of meshing impact and separation.

**Fig 12:**
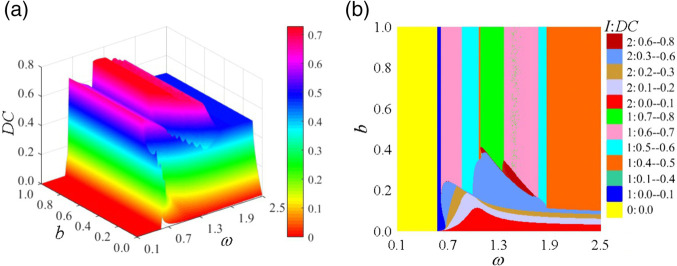
Impact states: disengagement duty cycle (I: DC) diagram in the (ω, b) parameter plane: (a) DC cloud diagram, (b) I: DC plane distribution diagram.

The *DC* cloud diagram of the curve-face gear transmission system in the (*ω*, *b*) parameter plane is depicted in Fig 12. Fig 12(a) illustrates the three-dimensional DC cloud diagram within the (*ω*, *b*) parameter plane. It is observed that when *b* > 0.4125, the system exhibits a non-impact meshed 0/1T periodic motion region with *DC* = 0.0. As *ω* increases, the system traverses the grazing bifurcation, where the impact state transitions from *I* = 0 to *I* = 1, resulting in an instantaneous increase in the *DC* value with a relatively small amplitude. Subsequently, the system undergoes a jump, which causes a drastic increase in *DC* to 0.6–0.7, indicating severe disengagement and a reduced load-bearing capacity. As *ω* continues to increase, the system enters quasi-periodic motion, during which *DC* gradually increases, resulting in worsening disengagement characteristics and load-bearing capacity. A further increase in *ω* drives the system into chaotic motion, where *DC* reaches its peak, corresponding to the most severe disengagement and weakest load-bearing capacity. As *ω* increases further, the system experiences an inverse period-doubling bifurcation, causing chaotic motion to degenerate into low-periodic motion. During this process, *DC* gradually decreases. Ultimately, a smooth *DC* surface is formed in the 1/1T periodic motion region. For *b*∈[0.4125, 1.0], in the bilateral impact state region, as *b* increases, the system transitions from single-periodic motion to high-periodic motion, accompanied by an increase in *DC*. As shown in Fig 12 (b), the (*ω*, *b*) parameter plane diagram of impact states (*I*: *DC*) illustrates the distribution range and variation pattern of disengagement duty cycles across different impact states. When *b* is large, *DC* exhibits a linear strip-like distribution. With increasing *ω*, the variation follows the sequence: 0:0.0 → 1:0.0–0.1 → 1:0.1–0.2 → 1:0.6–0.7 → 1:0.4–0.6 → 1:0.7–0.8 → 1:0.6–0.7 → 1:0.4–0.6. When *b* is small, the *DC* exhibits an increasing trend along both the *ω* and *b* axes.

**Fig 13:**
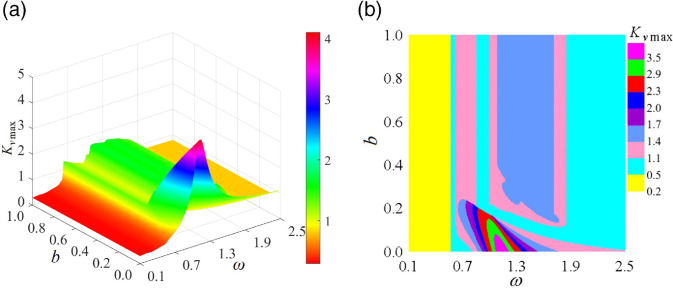
Maximum dynamic load coefficient $K_{vmax}$ in the (ω, b) parameter plane: (a) $K_{vmax}$ cloud diagram, (b) $K_{vmax}$ plane distribution diagram.

Fig 13 illustrates the maximum dynamic load $K_{vmax}$ of the curve-face gear transmission system within the (*ω*, *b*) parameter plane. Fig 13(a) presents a three-dimensional cloud diagram of $K_{vmax}$, where $K_{vmax}$ forms a dynamic load surface that gradually increases along the *ω*-axis in the 0/1T periodic motion region. It reaches its peak in the double-sided impact 2/1T motion region and subsequently decreases with increasing *b* when *b* is small. Fig 13(b) depicts the $K_{vmax}$ plane distribution diagram, revealing the variation pattern of $K_{vmax}$. As the system traverses the jump, $K_{vmax}$ experiences an instantaneous linear increase due to severe hard impacts on the gear pair. When *b* is large, increasing *ω* drives the system into quasi-periodic motion, where intensified vibrations and noise cause $K_{vmax}$ to rise again. Subsequent continuous period-doubling bifurcations lead to chaotic motion, during which $K_{vmax}$ rapidly peaks in the chaotic region. As *ω* continues to increase, chaotic motion degenerates into low-periodic motion, resulting in a decrease in $K_{vmax}$, forming a smooth dynamic load surface in the 1/1T periodic motion region.

### 3.3 Pattern types and transition laws of periodic motion in (*ω*omega, ξxi) parameter plane

**Fig 14:**
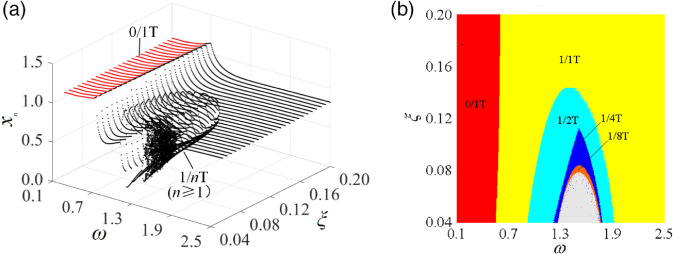
Meshing motion law diagram of the system in the (ω, ξ) parameter plane: (a) relative micro-displacement bifurcation diagram of gear pair, (b) pattern types and distribution regions of periodic motions.

With dimensionless meshing frequency (*ω*) and meshing damping ratio (ξ) serving as control parameters, where *ω* ∈ [0.1, 2.5] and ξ ∈ [0.04, 0.16], the nonlinear dynamic behavior of the curve-face gear transmission system is investigated within the (*ω*, ξ) parameter plane, while other parameters remain fixed. Fig 14 illustrates the distribution of meshing motion in this parameter space. Fig 14(a) presents a three-dimensional bifurcation diagram of meshing displacement against parameters (*ω*, ξ), indicating that non-impact motion predominates in the low-frequency region. At lower values of ξ, a jump occurs in the low-frequency region, transitioning the system from non-impact motion to tooth surface impact motion. In the high-frequency region, continuous period-doubling bifurcations propel the system into high-period motion, ultimately culminating in chaotic motion. Conversely, at larger values of ξ, the gear pair demonstrates single-period motion across all parameter ranges. Fig 14(b) depicts the meshing motion distribution map in the (*ω*, ξ) parameter plane, highlighting red regions of 0/1T non-impact motion, yellow regions of 1/1T tooth-surface impact motion, color-coded regions representing subharmonic motions, and gray regions indicating chaotic motion. The transition laws are as follows: in the low ξ, low-frequency region, the system exhibits 0/1T non-impact motion. As *ω* increases, grazing bifurcation occurs, transitioning from 0/1T non-impact motion to 1/1T tooth-surface impact motion. This is followed by a jump, which maintains the motion type. Further increases in *ω* trigger period-doubling bifurcations, transitioning from 1/1T motion to subharmonic motions (1/2T, 1/4T, 1/8T), ultimately leading to chaotic motion. Inverse period-doubling bifurcations gradually reduce chaotic motion into low-period motions (1/8T → 1/4T → 1/2T), eventually stabilizing into 1/1T periodic motion. Increasing ξ leads to an expansion of the 0/1T and 1/1T motion domains while shrinking the subharmonic motion domains. When ξ > 0.1436, only 0/1T and 1/1T meshing motions exist in the (*ω*, ξ) parameter plane. In conclusion, for systems with small ξ, a judicious increase in ξ effectively reduces chaotic motion.

**Fig 15:**
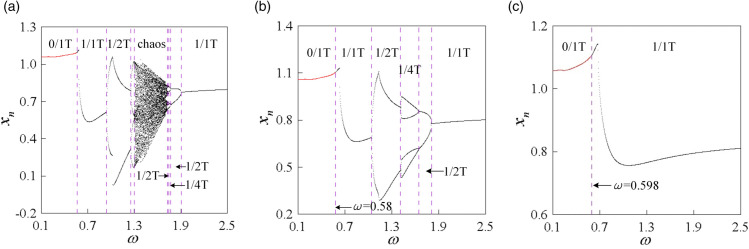
Single-parameter bifurcation diagrams of mesh displacement under different meshing damping ratios ξ: (a) ξ = 0.05, (b) ξ = 0.09, (c) ξ = 0.15.

Fig 15 illustrates the single-parameter bifurcation diagrams of the system for various values of ξ, clearly depicting the transition process among different types of periodic motion. It is evident that as ξ increases, the period-doubling bifurcation points progressively lag, while the inverse period-doubling bifurcation points advance. With the increase of both *ω* and ξ, and in conjunction with Fig 14, the fundamental laws governing the impact motion of the curve-face gear system within the (*ω*, ξ) parameter plane can be summarized as follows:

1. When ξ = 0.05, 0/1T → (G Bif) → 1/1T → (PD Bif) →1/2T → (PD Bif) →1/4T → (PD Bif) →... chaos... → (PD Bif) → 1/8T → (PD Bif) → 1/4T → (PD Bif) → 1/2T → (PD Bif) → 1/1T, Fig 15(a) shows.
2. When ξ = 0.09, 0/1T → (G Bif)→1/1T → (PD Bif) → 1/2T → (PD Bif) → 1/4T → (PD Bif) → 1/2T → (PD Bif) → 1/1T, Fig 15(b) shows.
3. When ξ = 0.15, 0/1T → (G Bif) → 1/1T, Fig 15(c) shows.

**Fig 16:**
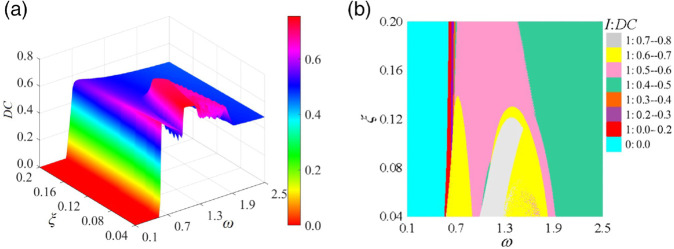
Impact states: disengagement duty cycle (I: DC) diagram in the (*ω*, ξ) parameter plane: (a) DC cloud diagram, (b) I: DC plane distribution diagram.

Fig 16 illustrates the *DC* cloud diagram and the distribution of impact states within the (*ω*, ξ) parameter plane. Fig 16(a) demonstrates the variation of *DC* with respect to ξ. For ξ < 0.1436, the system stabilizes into single-period motion with negligible variation in *DC*. Fig 16(b) emphasizes the evolution of impact states. An increase in ξ results in a delay of the jump points, thereby enhancing the critical speed threshold for tooth separation. Within the range ξ ∈ [0.04, 0.1436], the *DC* follows a complex trajectory along *ω*, marked by an increase, decrease, increase, and decrease. For ξ > 0.1436, *DC* evolves smoothly along the *ω*-axis, transitioning from 0:0.0 to 1:0.0–0.2, then to 1:0.2–0.3, followed by 1:0.3–0.4, and finally to 1:0.4–0.5, indicating a gradual rise.

**Fig 17:**
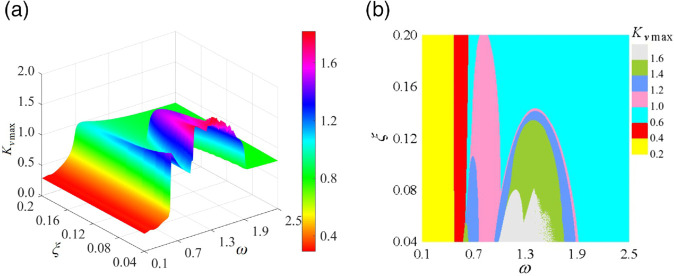
Maximum dynamic load coefficient $K_{vmax}$ in the (ω, ξ) parameter plane:(a) $K_{vmax}$ cloud diagram, (b) $K_{vmax}$ plane distribution diagram.

Fig 17 illustrates the $K_{vmax}$ of the curve-face gear transmission system within the (*ω*, ξ) parameter plane. In Fig 17(a), the relationship between $K_{vmax}$ and control parameters is presented. In the low-frequency 0/1T motion region, $K_{vmax}$ forms a continuously rising surface along the *ω*-axis. Upon crossing a grazing bifurcation or jump, $K_{vmax}$ experiences abrupt changes; notably, it increases linearly for small values of ξ. In the subharmonic motion region, $K_{vmax}$ manifests as a tongue-shaped plateau, with the plateau height decreasing as ξ increases. Fig 17(b) displays the contour map of $K_{vmax}$. For small ξ, during the jump, $K_{vmax}$ spikes to between 1.8 and 2.1, indicating a significant impact of loads on the gear pair. As ξ increases, the impact magnitude during the jump gradually diminishes. In the subharmonic region, $K_{vmax}$ demonstrates a gradient decrease along the ξ-axis.

## 4 Conclusion

This study established a six-degree-of-freedom bending-torsion coupled nonlinear dynamic model of curve-face gear transmission systems, which comprehensively considers multiple nonlinear factors such as tooth surface friction, static transmission error, tooth side clearance, and time-varying stiffness. Based on the two-parameter co-simulation numerical method, the pattern types and existence regions of the periodic motions in the two-parameter plane formed by key parameters were investigated. In combination with bifurcation diagrams, duty cycle diagrams, dynamic load diagrams, phase diagrams, and spectrum diagrams, the transition mechanisms between non-impact vibration and tooth impact vibration, as well as between adjacent basic periodic motions, were systematically revealed. Additionally, the effects of parameter variations on the types and existence regions of periodic motion patterns were analyzed.

1. Under the baseline parameters, the gear system operates in a non-impact state when *ω* < 0.569. As *ω* increases, the system transitions through grazing bifurcation into tooth surface impact 1/1T motion, followed by continuous period-doubling bifurcations into chaotic motion, ultimately returning to 1/1T motion via inverse period-doubling bifurcations. The transition from non-impact 0/1T motion to tooth surface impact 1/1T motion is accomplished through grazing bifurcation. The transitions between adjacent tooth surface impact motions, 1/*n*T motions, are achieved via period-doubling bifurcation.
2. In the (*ω*, *b*) parameter plane, small values of *b* induce double-sided impact states characterized by low dynamic coefficients but severe impact loads, resulting in intense vibrations and noise, as well as reduced stability. An increase in *ω* diminishes the double-sided impact motion, lowers the maximum dynamic load coefficient ($K_{vmax}$), and stabilizes the system. For the range 0.4125 ≤ *b* ≤ 1.0, variations in *b* do not alter the periodic motion pattern but only affect meshing displacement, with key bifurcation thresholds (grazing, jump, period-doubling, inverse period-doubling) and dynamic coefficients remaining unchanged, resembling a linear system.
3. In the (*ω*, ξ) parameter plane, small values of ξ lead to complex dynamic behaviors. As ξ increases, the number of periodic motion modes decreases, while the regions of 0/1T and 1/1T motion expand, causing other motion regions to shrink or disappear; the *DC* and $K_{vmax}$ decline. Additionally, the point of grazing bifurcation is delayed. Consequently, increasingξimproves motion characteristics, postpones the jump, and promotes operational smoothness.

Under extreme operating conditions, gear transmission systems are prone to exhibiting nonlinear dynamic phenomena such as jump vibrations, double-sided impact, meshing instabilities, and chaotic motions. By optimizing the design parameter configurations of gear pairs, it is possible to effectively suppress the unstable vibration regions of the system while significantly reducing the dynamic instability issues caused by excessive meshing impacts.

## Limitations and deficiencies

This paper investigates the nonlinear dynamic characteristics of the curve-face gear transmission system using a two-parameter co-simulation method. However, several key issues remain to be addressed in future research. Therefore, the next phase will further explore the effects of friction, gear wear, and manufacturing tolerances on the model’s performance, aiming to enhance the robustness and reliability of the model in practical engineering applications.
